# The long-term negative impact of childhood stroke on language

**DOI:** 10.3389/fped.2024.1338855

**Published:** 2024-05-07

**Authors:** Magdalena Heimgärtner, Alisa Gschaidmeier, Lukas Schnaufer, Martin Staudt, Marko Wilke, Karen Lidzba

**Affiliations:** ^1^ Department of Pediatric Neurology and Developmental Medicine, University Children’s Hospital, Tübingen, Germany; ^2^ Center for Pediatric Neurology, Neurorehabilitation and Epileptology, Schön Klinik Vogtareuth, Vogtareuth, Germany; ^3^ Division of Neuropediatrics, Development and Rehabilitation, University Children’s Hospital Inselspital, Bern University Hospital, University of Bern, Bern, Switzerland; ^4^ Experimental Pediatric Neuroimaging, Children’s Hospital and Department of Neuroradiology, University Hospital Tübingen, Tübingen, Germany; ^5^ Center for Pediatric Palliative Care, Dr von Hauner Children’s Hospital, University of Munich, Munich, Germany

**Keywords:** childhood stroke, aphasia, chronic language deficits, unilateral brain lesion, perinatal stroke

## Abstract

**Objectives:**

This study aims to investigate the long-term language outcome in children with unilateral childhood stroke in comparison to those with perinatal strokes and typically developing individuals and to explore the impact of lesion-specific modifiers.

**Methods:**

We examined nine patients with childhood stroke, acquired between 0;2 and 16;1 years (CHILD; 3 female, median = 13.5 years, 6 left-sided), 23 patients with perinatal strokes (PERI; 11 female, median = 12.5 years, 16 left-sided), and 33 age-matched typically developing individuals (CONTROL; 15 female, median = 12.33 years). The language outcome was assessed using age-appropriate tasks of the Potsdam Illinois Test of Psycholinguistic Abilities (P-ITPA) or the Peabody Picture Vocabulary Test (PPVT). For group comparisons, study-specific language *z*-scores were calculated. Non-verbal intelligence was assessed using the Test of Non-verbal Intelligence (TONI-4), language lateralization with functional MRI, and lesion size with MRI-based volumetry.

**Results:**

All four patients with childhood stroke who initially presented with aphasic symptoms recovered from aphasia. Patients with childhood stroke showed significantly lower language scores than those in the control group, but their scores were similar to those of the patients with perinatal stroke, after adjusting for general intelligence (ANCOVA, language *z*-score CHILD = −0.30, PERI = −0.38, CONTROL = 0.42). Among the patients with childhood stroke, none of the possible modifying factors, including lesion side, correlated significantly with the language outcome.

**Conclusion:**

Childhood stroke, regardless of the affected hemisphere, can lead to chronic language deficits, even though affected children show a “full recovery.” The rehabilitation of children and adolescents with childhood stroke should address language abilities, even after the usually quick resolution of clear aphasic symptoms.

## Introduction

In adults, aphasia is a common symptom of stroke, affecting approximately 30% of all patients being affected (1, 2). It is more common in patients who had left hemispheric stroke (1), and those with exclusively hemorrhagic etiology have a slightly lower incidence than that of those with ischemic or mixed etiologies (1). In particular, lesions to the superior temporal lobe are associated with persistent aphasia syndromes (3). Data on recovery from aphasia is limited. Typically, aphasia often is a persisting impairment with less than 24% of the affected patients fully recovering within 18 months (1). Although most patients show signs of recovery during the first 6 months after stroke (3), improvement can be observed until much later (3, 4).

After perinatal stroke, language development may be delayed, and compared to healthy controls, children after perinatal stroke often score significantly lower on standardized language tests well into middle childhood (5–7). A large part of this effect, however, can be attributed to epilepsy (5, 8). In children without epilepsy, often only subtle deficits in language function are detected (9, 10), if any (11).

Language development after perinatally acquired left hemispheric lesions is probably the most prominent example of early brain plasticity. Contrary to recovery from aphasia in adults, which heavily relies on the perilesional remainders of the left hemispheric language network (12), language development after perinatal left hemispheric brain lesions is often associated with atypically right hemispheric language representation (11, 13–16). The driving factors for inter- vs. intrahemispheric language representation in this patient group are still subject to research (7, 17). Of note, this compensatory hemispheric reorganization is not always better: in smaller left hemispheric lesions, typical language representation might be associated with better functional outcomes than if atypical language representation ensues (6).

Ischemic stroke occurring after the neonatal period (“childhood stroke”) has an incidence of 1–3 in 100,000 children per year (18, 19). It is therefore rarer than perinatal stroke (4 in 10.000 live births per year (19), despite the neonatal period lasting only 4 weeks. General cognitive functioning after such incidents seems to be less favorable when the stroke happened early as compared to late childhood (20, 21); however, the relationship between age at insult and outcome is probably non-linear (22). In comparison to healthy controls, children after childhood stroke score lower in expressive and receptive language tests (15). Data on aphasia resulting from childhood stroke is limited and is complicated by the fact that even the definition of aphasia in children is not universally agreed upon (23). Although many clinicians would probably agree that aphasia after stroke is less common in children than in adults, the limited data available suggests that, as in adults, about one-third of all childhood stroke incidents seem to result in aphasic symptoms (23). With regard to recovery, however, the natural course in children and adolescents is better (24): 6 months after an acquired brain injury, only a small fraction of children still show severe language problems, much less than in a comparable cohort of young adults (25). Interhemispheric language reorganization does not seem to be a common compensatory mechanism in childhood stroke, since most affected individuals have typical left hemispheric representation (15). Right-sided language representation is significantly associated with younger age at injury (24, 26). Due to small sample sizes, data on the quality of language recovery from aphasia after childhood brain lesions is limited, and the moderating factors remain unclear.

In this retrospective cross-sectional observation study, we explored the long-term language outcome after unilateral brain lesions acquired during childhood and adolescence. Patients with perinatally acquired unilateral brain lesions and typically developing individuals without brain lesions were included as control groups.

Although stroke is often considered less disabling in children than in adults, we hypothesized that individuals with childhood lesions (CHILD) would have worse language function than healthy controls (CONTROL) of the same age (H1). In comparison to the patient control group with perinatal strokes (PERI), we also expected worse language outcome (H2).

## Materials and methods

### Subjects

Participants were recruited in two large neuropediatric centers in Germany (University Children's Hospital Tübingen and Schön Klinik Vogtareuth) by searching the clinical database, by personal contacts, and during hospitalization, using the same in- and exclusion criteria. Typically developing controls were recruited from the general population through advertisements in the local press and the clinic’s internal information system. After they contacted our study staff members, they were screened using a questionnaire asking for any neurological or psychiatric diagnosis and problems in cognitive or language development. A formal assessment confirming normal development was not used.

General inclusion criteria were German as a native language and age ≥ 8 years at study participation. The general exclusion criteria included contraindications for MRI, a previous neurological or psychiatric diagnosis (apart from the brain lesion in the patient groups), and a previous diagnosis of intellectual disability (IQ below 70). Therefore, we relied on the medical history or the self-reported intelligence scores. Additionally, standardized non-verbal IQ scores were acquired during the study procedure from the Test of Non-verbal Intelligence—Fourth Edition (TONI-4), and participants with a non-verbal IQ below 70 were excluded.

The CHILD group included nine patients (three female, median age = 13.5 years, range 8–27 years, six left-sided lesions) with unilateral brain lesions such as ischemic or hemorrhagic infarctions acquired at the age between 29 days and 18 years and at least 1 year before study participation.

The PERI group (patient control group) included 23 patients with perinatally acquired unilateral brain lesions such as ischemic or hemorrhagic infarctions at an age before 29 days (11 female, median age = 12.5 years, range 8–26 years, 16 left-sided lesions).

The CONTROL group included 33 typically developing individuals (15 female, median age = 12.33, range 8–29 years).

The majority of the children in the patient groups attended a regular school. Five children attended a school for children with special needs, while six children attended a regular school with a school escort providing support, mostly due to their motor impairment. Data about school type was missing in two cases.

The study was approved by the local ethics committee at the University Hospital Tübingen (No. 693/2014B01). All adult participants and the parents of underage participants gave their written, informed consent. The study was performed in accordance with the Code of Ethics of the World Medical Association (Declaration of Helsinki in its latest implementation).

### Structural and functional MRI

The participants were examined with a Siemens 1.5 T Avanto (Tübingen) or Symphony (Vogtareuth) MRI scanner. Functional and anatomical images were analyzed with SPM12 (Statistical Parametric Mapping; Wellcome Department of Imaging Neurosciences, UCL, UK), CAT12 (by Christian Gaser and Robert Dahnke, Departments of Psychiatry and Neurology, Jena University Hospital), and Matlab (MathWorks, Natick MA, USA). Lesion size was determined with a semiautomated approach (27).

Language lateralization was determined by fMRI using the vowel identification task (17, 28, 29). More information about the MRI sequences and data processing and analysis steps can be found in (30).

### Neuropsychological protocol

To assess language abilities, participants completed two different tests: the German version of the Peabody Picture Vocabulary Test-III (PPVT) and the German version of the Potsdam Illinois Test of Psycholinguistic Abilities (P-ITPA). The PPVT measures the subject's receptive vocabulary ability (31). German standardized age norms cover the range from 13 to 71 years. The P-ITPA (Language Development Score) is a more comprehensive battery containing measurements of verbal intelligence, expressive vocabulary, expressive language skills, phonological awareness, and verbal short-term memory (32). Standardized age norms cover the range from 4;0 to 11;6 years. For participants aged 11;6 years or younger, the appropriate age norms of the P-ITPA were applied, and for participants aged 13;0 years or older, the appropriate age norms of the PPVT were applied. One patient and three controls were out of the age ranges of both tests (11;8–12;10 years old). Here, we used the oldest group of P-ITPA for the two subjects aged <12 years and the youngest group of the PPVT for the two subjects aged >12 years.

For the assessment of non-verbal intelligence, we used the TONI-4, a motor-independent intelligence test that measures the ability for abstract reasoning. Standardized age norms cover the range from 6;0 to 89;11 years (33).

### Statistical analysis

Within our sample, the raw scores of P-ITPA and PPVT were highly correlated (r = .845; 95% CI [.756,.903). Since the actuality of standardization was strongly divergent between the tests, we subjected the age-appropriate *T*-scores (for each participant either P-IPTA or PPVT) and index scores (TONI-4) of the participants to a study-specific *z*-transformation. The resulting *z*-score of the age-appropriate language test (either P-ITPA or PPVT) was then transferred to the new variable “language score.”

We then conducted a univariate analysis of covariance (ANCOVA), with the factor group (CHILD vs. PERI vs. CONTROL) and the independent variable “language score” (either P-ITPA or PPVT sample-standardized *z*-score, according to age). We introduced the covariates “language test” (i.e., the use of P-ITPA or PPVT) and IQ (TONI-4 *z*-score).

Since epilepsy was present only in the PERI group, we were not able to use epilepsy as a covariate. Thus, we repeated the same analysis with only those PERI patients without epilepsy *post hoc*.

To identify potential modifiers of language performance in the patient groups, we conducted separate correlation analyses between the variable “language score” and sex (male vs. female), age at injury, intelligence (TONI-4), lesion side (left vs. right), lesion volume (vol), and language lateralization index (LI). The significance threshold was Bonferroni-corrected for multiple tests, resulting in a threshold of *p* = 0.05/6 = .0083.

## Results

Individual patient characterization can be found in Table 1. Group comparisons are shown in Table 2.

**Table 1:** Patient characteristics.

Patient code	Sex	Age at scan (year;month)	Age at injury (year;month)	Lesion side	Lesion size (cm3)	Lesion type	Initial aphasia	Epilepsy	LI	TONI-4 *z*-score	PPVT *z*-score	P-ITPA *z*-score	Language *z*-score
Group CHILD													
* T43*	male	8;2	0;2	Right	1.31	HS	N/A	No	.78	0.40	N/A	0.20	0.20
* T40*	female	24;6	9;2	Left	2.28	AIS	Yes	No	.85	−0.07	−0.40	N/A	−0.40
* T51*	male	8;0	3;1	Left	12.95	AIS	Yes	No	N/A	−1.07	N/A	−1.00	−1.00
* T55*	male	10;10	6;5	Left	14.61	HS	No	No	.82	0.60	N/A	1.30	1.30
* T10*	female	14;8	7;10	Left	44.22	AIS	Yes	No	.74	0.93	2.30	N/A	2.30
* V07*	female	13;6	11;8	Right	107.05	HS	No	No	.75	−0.07	0.60	N/A	0.60
* V05*	male	19;1	16;1	Left	108.46	AIS	Yes	No	.73	1.47	0.00	N/A	0.00
* T08*	male	27;7	13;10	Right	117.37	HS	No	No	.92	0.33	−0.20	N/A	−0.20
* V10*	male	8;6	4;10	Left	141.65	HS	No	No	.70	−0.07	N/A	−1.20	−1.20
Group PERI													
* T28*	male	16;9	0	Left	N/A	Not classified^a^	N/A	Yes	.69	−1.10	0.09	N/A	0.09
* V04*	female	15;7	0	Right	N/A	PVI	N/A	No	N/A	−1.01	0.01	N/A	0.01
* T57*	female	19;5	0	Left	1.16	PVI	N/A	No	.64	−0.82	−0.52	N/A	−0.52
* V09*	female	10;10	0	Left	1.24	PVI	N/A	No	N/A	0.61	N/A	0.62	0.62
* T56*	male	12;10	0	Right	9.08	PVI	N/A	No	.55	0.70	−1.85	N/A	−1.85
* T13*	male	8;10	0	Left	22.82	PVI	N/A	Yes^!^	.85	−0.25	N/A	−1.18	−1.18
* V01*	male	8;9	0	Left	24.80	PVI	N/A	No	.61	−0.15	N/A	−0.98	−0.98
* V06*	male	8;0	0	Left	28.83	PVI	N/A	No	.78	−0.82	N/A	0.02	0.02
* T21*	female	23;7	0	Left	29.57	PVI	N/A	No	−.25	−0.91	−0.70	N/A	−0.70
* T19*	female	23;7	0	Left	36.05	PVI	N/A	No	−.83	−0.25	−1.32	N/A	−1.32
* V08*	female	11;5	0	Left	41.65	AIS	N/A	No	−.53	1.84	N/A	−0.18	−0.18
* V02*	female	9;2	0	Right	45.11	PVI	N/A	Yes	N/A	0.23	N/A	−1.18	−1.18
* V11*	male	10;0	0	Left	50.29	PVI	N/A	No	.63	−1.10	N/A	−0.78	−0.78
* V03*	female	11;9	0	Left	55.82	PVI	N/A	No	.67	−0.44	N/A	0.72	0.72
* V14*	male	10;6	0	Right	79.38	PVI	N/A	No	N/A	−0.82	N/A	−0.18	−0.18
* T44*	male	16;8	0	Right	84.97	AIS	N/A	Yes	.87	−1.39	−1.49	N/A	−1.49
* V13*	female	10;1	0	Left	85.36	PVI	N/A	No	−.31	0.04	N/A	0.52	0.52
* T12*	female	12;6	0	Left	97.30	PVI	N/A	No	−.32	0.70	0.27	N/A	0.27
* T14*	male	16;1	0	Left	133.93	AIS	N/A	Yes^!^	−.50	−2.34	−2.02	N/A	−2.02
* T33*	male	23;7	0	Left	161.77	PVI	N/A	No	−.66	−0.44	−0.97	N/A	−0.97
* T39*	male	26;1	0	Left	167.79	AIS	N/A	Yes	−.87	−1.39	−0.97	N/A	−0.97
* T41*	male	22;0	0	Right	200.57	PVI	N/A	Yes	.89	−1.20	−1.23	N/A	−1.23
* V12*	female	10;1	0	Right	220.71	PVI	N/A	Yes^!^	−.53	−0.06	N/A	−0.08	−0.08

AIS, arterial ischemic stroke; HS, hemorrhagic stroke; LI, lateralization index; PVI, periventricular venous infarction; T, Tübingen; V, Vogtareuth.

^a^ Lesion could not be classified as AIS or PVI because only an MRI after implantation of a ventriculoperitoneal shunt was available.

^!^ Drug-resistant epilepsy.

**Table 2:** Sample characterization.

	CHILD (*n* = 9)	CONTROL (*n* = 33)	PERI (*n* = 23)	Significance
Age at MRI [months; median (range)]	162 (96–331)	148 (98–353)	150 (96–313)	*p* = .929^a^
Male/female	6/3	18/15	12/11	*χ*^2^ = .752
TONI-4 index score [IQ-scale; median (range)]	105 (84–122)	103 (82–124)	97 (77–121)	*p* = .024^a^
P-ITPA index score [T-scale; median (range)]	*n* = 4 46 (38–63)	*n* = 16 56 (41–80)	*n* = 11 51 (41–60)	*p* = .142^a^
PPVT index score [*T*-scale; median (range)]	*n* = 5 54 (48–73)	*n* = 17 63 (51–73)	*n* = 12 45 (33–59)	*p* < .001^a^

^a^ Non-parametrical median comparison (Kruskal–Wallis), two-tailed.

CHILD group: Six out of nine patients had a left-sided stroke. Of these, four initially presented with aphasia from which all had recovered at the time of study. No patient in this group had epilepsy. In one child with a left-sided stroke, the functional MRI activation was not strong enough for interpretation of the language lateralization score. The remainder of the group showed a left-sided activation pattern.

PERI group: 8 out of 23 patients had epilepsy (three drug-resistant), and 16/23 patients had a left-sided lesion. Of these, seven had a typical left-sided language representation and eight had an atypical pattern (one patient without interpretable fMRI). Of the nine patients with right-sided lesions, one had an atypical language activation pattern.

Language lateralization differed significantly between the three groups (ANOVA, F_2,57_ = 18.093, *p* < .001). *Post hoc* Scheffé tests revealed that group PERI had significantly less leftward-oriented language than the other two groups (mean LI CHILD = .786, mean LI PERI = .125, mean LI CONTROL = .772; *p* < .001). Also, non-verbal intelligence differed significantly between the three groups (ANOVA, F_2,62_ = 3.912, *p* = .025). *Post hoc* Scheffé tests revealed that group PERI scored significantly lower than group CONTROL, but not significantly lower than group CHILD (mean TONI-z CHILD = 0.238, mean TONI-z PERI = −0.45, mean TONI-z CONTROL = 0.253, *p* = .033).

**Figure 1:**
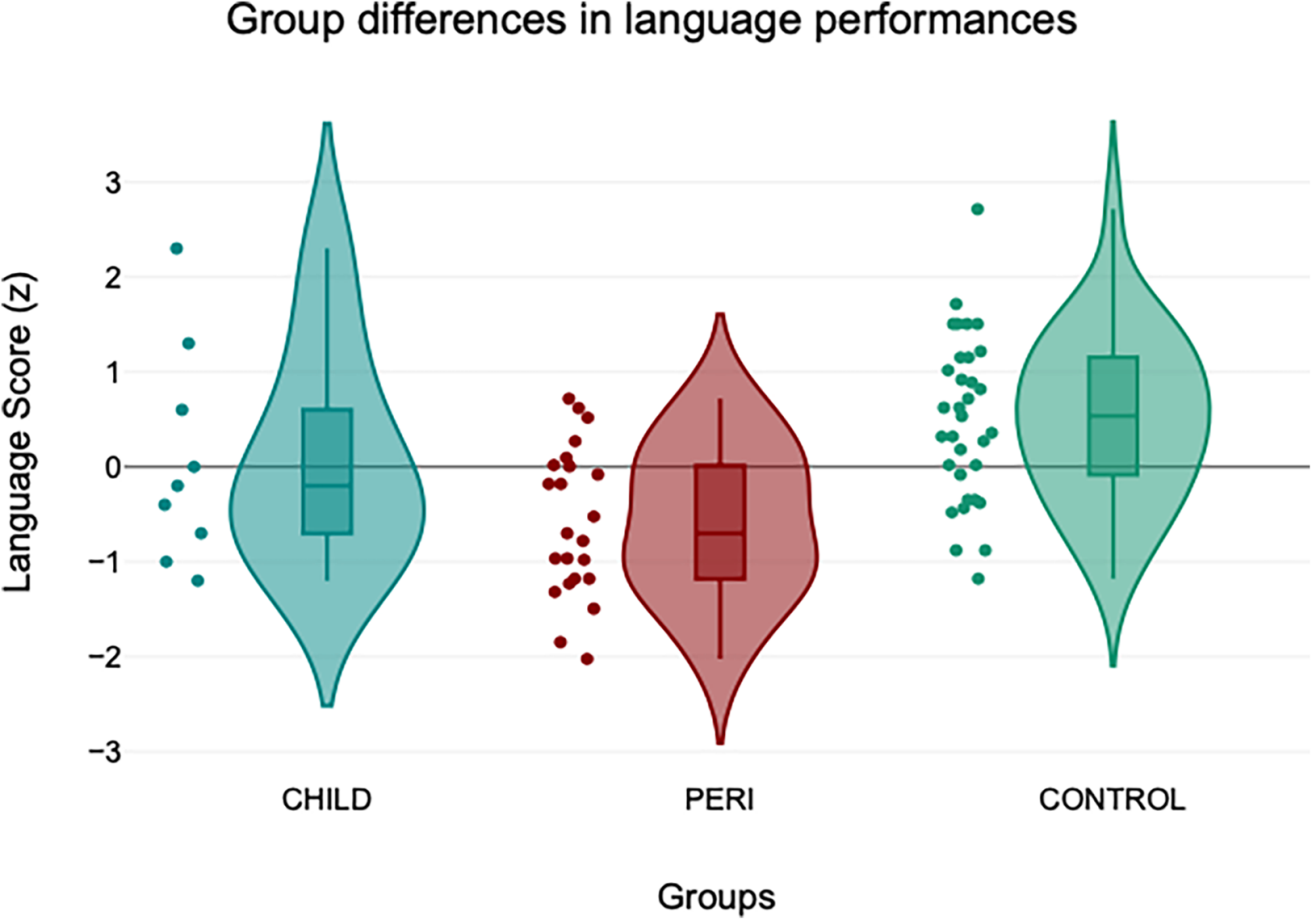
Group differences in language performance.

Testing our main hypothesis, the ANCOVA revealed a significant group effect for language score, after correcting for language test version and intelligence (F_2,60_ = 8.059; *p* = .001). Both patient groups scored significantly lower than the control group (mean corrected language score CHILD = −0.299, mean corrected language score PERI = −0.378, mean corrected language score CONTROL = 0.410), as depicted in Figure 1.

After the exclusion of patients with epilepsy from the PERI group, the results remained largely the same: A significant group effect on language score (F_2,45_ = 6.701; p = .003), with both patient groups scoring significantly lower than the control group (mean corrected language score CHILD = −0.237, mean corrected language score PERI = −0.214, mean corrected language score CONTROL = 0.451).

For group CHILD, none of the potential modifiers lesion side, sex, lesion size, age at the time of injury, or time since the event correlated significantly with language scores (Table 3A). In group PERI, language performance was significantly associated with sex (*r* = −0.517, *p* = .012), but not with any of the other variables (Table 3B).

**Table 3:** Correlation coefficients between potential modifiers and language performance in patients with postneonatal childhood stroke (A) and patients with perinatal stroke (B).

*z*-score language performance	Lesion side	Sex	Lesion size	Age at injury	Time since injury	Lateralization index
A CHILD group ^a^ (*N* = 9)	*τ* = −.079, *p* = .796	*τ* = .471, *p* = .121	*τ* = −.333, *p* = .211	*τ* = .000, *p* = 1.000	*τ* = .000, *p* = 1.000	*τ* = .000, *p* = 1.000
B PERI group^b^ (*N* = 23)	*r* = −.238, *p* = .273	*r* = −.517, *p* = .012	*r* = −.141, *p* = .531	N/A	N/A	*r* = −.005, *p* = .985 (*N* = 19)

^a^ Non-parametrical correlations (Kendall’s tau-b), two-tailed.

^b^ Parametrical correlations (Pearson *r*), two-tailed.

## Discussion

Our main finding is that unilateral brain lesions acquired in childhood and adolescence can lead to chronic language deficits, regardless of the hemisphere affected. This result is both surprising and of clinical relevance.

Language reorganization after left hemispheric lesions to the developing brain (with, unlike in adults, the right hemisphere has the potential to “take over”) is probably the best-known example of the special compensatory potential of a child's central nervous system (“developmental plasticity”). Many studies have demonstrated that *pre- and perinatally acquired* left hemispheric brain lesions often lead to atypical right hemispheric language representation (13, 15, 16, 34, 35), a compensatory mechanism that does obviously not belong to the repertoire of a mature brain. Atypical right hemispheric language representation is probably the reason for the relatively good language outcome in these individuals, which is clearly better than the chronic aphasia seen in adults with comparable brain lesions (36). However, there is also broad evidence that a perinatally acquired brain lesion can impair language function when compared to that of individuals with non-lesioned brains (5, 22, 37–39), although the presence of epilepsy may be a decisive factor (8). The fact that we now show here that individuals with a history of unilateral brain lesions acquired in childhood, and without epilepsy, perform below healthy controls in language tests, therefore is unexpected.

Due to the low incidence of childhood stroke, data on language outcome of these patients is only just accumulating. Post-stroke acquired aphasia affects approximately one-third of childhood stroke patients [here defined as “acquired after the age of two years” (23)] and is therefore as common in children and adolescents as it is in adults (1). However, younger individuals seem to fully recover more often from stroke-induced aphasia than older ones (40). Indeed, while a substantial proportion of adults have aphasic symptoms years after the incident, chronic aphasia is extremely rare in children and adolescents. The neurological mechanism underlying this recovery is different from the one following lesions acquired in the neonatal period since successful *inter*hemispheric reorganization of language is uncommon following brain lesions acquired after the age of two to three years (15, 24). This was the rationale for our hypothesis that patients with perinatal stroke would have better language function than patients with postneonatal stroke. However, this was not the case: While both patient groups had significantly lower language scores than healthy controls after correcting for general intelligence, no significant difference was detectable between the two patient groups (PERI and CHILD). Although the absence of significance is not evidence for equality, Figure 1 illustrates a striking overlap between the two groups. After controlling for the effect of epilepsy by excluding patients with epileptic seizures from the perinatal group, the groups converged even more. Thus, our results imply that a complex function such as language depends on an intact central nervous system.

Non-verbal fluid reasoning, however, seems to be more robust. In a previous study focusing on patients with perinatal brain lesions with or without epilepsy, we did not detect a significant difference in verbal function between healthy controls and patients with left hemispheric lesions without epilepsy (8). Our current results seem to contradict this finding, since both our patient groups differed significantly from controls in their language scores. As illustrated in Table 2, the controls' non-verbal intelligence lies within the middle normal range, arguing against a recruitment bias. A reason for the different results could, however, be our approach to language testing. In the previous study, we analyzed raw scores of the P-ITPA test for all participants and corrected for age in the analyses (8). For the current study, we used age-appropriate standard scores for P-ITPA or PPVT. Therefore, while sacrificing the advantage of homogeneous sets of tasks for all participants, we gained the advantage of acquiring age-appropriate standard scores for all participants. In *post hoc* analyses (available in the Supplementary Material), the significant difference between controls and patients with perinatal brain lesions is driven by the PPVT scores used for individuals older than 12 years. Interestingly, when analyzing the PPVT raw scores in the younger sample, no group effect can be identified, while an analysis of the P-ITPA raw scores for the older sample only reveals a significant group effect, even after excluding the patients with epilepsy. Thus, the older patients with perinatal lesions seem to have more prominent language difficulties than the younger ones, irrespective of the test used. This phenomenon of “growing into deficit” during late childhood and adolescence has been described before for general cognitive abilities in patients with neonatal stroke (41). In the future, systematically assessing older children and adolescents may help correct the picture that no (language or cognitive) deficits ensue following childhood stroke.

An additional conclusion to be drawn from our results is that lesion side does not seem to have a significant impact on language function in the chronic phase of recovery. This is all the more interesting as all patients with acute aphasia had a left hemispheric lesion, implying that the likely bilateral networks necessary to support complex functions such as language require sufficient neural substrate to develop.

The clinical impact of the long-term language impairment in both our patient groups is substantial. Although children and adolescents usually do recover from aphasia, the more subtle language problems we have identified here may significantly impair both the academic and social participation of the individuals, especially if the deficits become more obvious during late childhood. It was previously shown that in school-aged children, language proficiency significantly predicts reading abilities, secondary school achievement, and later occupational attainment (42). Language impairment may therefore critically influence social interaction and participation both in the family setting and, even more, in extrafamilial contexts (43). Thus, rehabilitation of children and adolescents with stroke should always keep an eye on language skills, even after clear aphasic symptoms have long since resolved.

### Limitations

The main limitation of our study remains the small sample size (CHILD, *n* = 9; PERI, *n* = 23; CONTROL, *n* = 33), which reduces statistical power and makes conclusions less generalizable. It must be considered, however, that childhood stroke is a rare event (18). Therefore, the recruitment of these patients is difficult and studies including a sufficiently large number of individuals for fine-grained analyses of modulating factors and interactions are, unfortunately, very difficult to conduct. Our results have therefore been considered as “preliminary.” The long-term language outcome should be investigated further in the future—ideally as multicenter studies and with the aid of more homogenous standardized tests. The need for two different language tests, due to the age of the participants, is another limitation of our study. However, in the absence of a standardized language test suitable for German-speaking individuals between 8 and 30 years, we had to use two different assessments. In our current analyses, we traded test homogeneity for age appropriateness, leading to a different perspective on the group comparisons than in a previous study using a different approach. Another limitation of our study is that there was no additional evaluation of psychiatric components and neurodevelopmental disorders carried out by a child neuropsychiatrist to guarantee the exclusion criteria. However, the existing medical records of the clinical databases were screened for psychiatric and neurological comorbidities for the patient group and, if available, previously performed neuropsychological tests were considered. A further limitation is that we have not collected data on learning disorders. Hence, we cannot exclude that despite average scores in non-verbal cognitive abilities, our patients show deficits in these aspects of cognition. Also, we have not collected data on the sociocultural and family context of our participants and therefore cannot exclude that they differ in the sociocultural and family context.

## Conclusion

We demonstrate that in childhood stroke (and similar to perinatally acquired stroke), lesion side does not clearly predict later language impairment, in agreement with previous studies (8, 44). Further, language impairment may only become obvious long after the initial insult, likely reflecting a “growing into deficit” mechanism. We therefore suggest following up language function not only in left hemispheric but also in right hemispheric childhood stroke and to continue to do so even after initial aphasic symptoms have resolved.

## Data Availability

The raw data supporting the conclusions of this article will be made available by the authors, without undue reservation.

## References

[1] Flowers HL Skoretz SA Silver FL Rochon E Fang J Flamand-Roze C Poststroke aphasia frequency, recovery, and outcomes: a systematic review and meta-analysis. Arch Phys Med Rehabil. (2016) 97(12):2188–201. e2188. 10.1016/j.apmr.2016.03.006 27063364

[2] Grönberg A Henriksson I Stenman M Lindgren AG. Incidence of aphasia in ischemic stroke. Neuroepidemiology. (2022) 56(3):174–82. 10.1159/000524206 35320798

[3] Gerstenecker A Lazar RM. Language recovery following stroke. Clin Neuropsychol. (2019) 33(5):928–47. 10.1080/13854046.2018.1562093 30698070 PMC8985654

[4] Martin KC Ketchabaw WT Turkeltaub PE. Plasticity of the language system in children and adults. Handb Clin Neurol. (2022) 184:397–414. 10.1016/B978-0-12-819410-2.00021-7 35034751 PMC10149040

[5] Ballantyne AO Spilkin AM Hesselink J Trauner DA. Plasticity in the developing brain: intellectual, language and academic functions in children with ischaemic perinatal stroke. Brain. (2008) 131(Pt 11):2975–85. 10.1093/brain/awn176 18697910 PMC2577808

[6] Beharelle AR Dick AS Josse G Solodkin A Huttenlocher PR Levine SC Left hemisphere regions are critical for language in the face of early left focal brain injury. Brain. (2010) 133(Pt 6):1707–16. 10.1093/brain/awq104 20466762 PMC2912693

[7] Ilves N Männamaa M Laugesaar R Ilves N Loorits D Vaher U Language lateralization and outcome in perinatal stroke patients with different vascular types. Brain Lang. (2022) 228:105108. 10.1016/j.bandl.2022.105108 35334446

[8] Gschaidmeier A Heimgärtner M Schnaufer L Driever PH Wilke M Lidzba K Cognitive development after perinatal unilateral infarctions: no evidence for preferential sparing of verbal functions. Eur J Paediatr Neurol. (2022) 37:8–11. 10.1016/j.ejpn.2021.12.007 34999444

[9] Schwilling E Krägeloh-Mann I Konietzko A Winkler S Lidzba K. Testing the language of German cerebral palsy patients with right hemispheric language organization after early left hemispheric damage. Clin Linguist Phon. (2012) 26(2):135–47. 10.3109/02699206.2011.595525 21787139

[10] Knecht M Lidzba K. Processing verbal morphology in patients with congenital left-hemispheric brain lesions. Brain Lang. (2016) 157-158:25–34. 10.1016/j.bandl.2016.04.011 27156034

[11] Newport EL Seydell-Greenwald A Landau B Turkeltaub PE Chambers CE Martin KC Language and developmental plasticity after perinatal stroke. Proc Natl Acad Sci U S A. (2022) 119(42):e2207293119. 10.1073/pnas.2207293119 36215488 PMC9586296

[12] Saur D Lange R Baumgaertner A Schraknepper V Willmes K Rijntjes M Dynamics of language reorganization after stroke. Brain. (2006) 129(Pt 6):1371–84. 10.1093/brain/awl090 16638796

[13] Rasmussen T Milner B. The role of early left-brain injury in determining lateralization of cerebral speech functions. Ann N Y Acad Sci. (1977) 299:355–69. 10.1111/j.1749-6632.1977.tb41921.x 101116

[14] Guzzetta A Pecini C Biagi L Tosetti M Brizzolara D Chilosi A Language organisation in left perinatal stroke. Neuropediatrics. (2008) 39(3):157–63. 10.1055/s-0028-1085465 18991195

[15] Ilves P Tomberg T Kepler J Laugesaar R Kaldoja ML Kepler K Different plasticity patterns of language function in children with perinatal and childhood stroke. J Child Neurol. (2014) 29(6):756–64. 10.1177/0883073813489350 23748202 PMC4230975

[16] Szaflarski JP Allendorfer JB Byars AW Vannest J Dietz A Hernando KA Age at stroke determines post-stroke language lateralization. Restor Neurol Neurosci. (2014) 32(6):733–42. 10.3233/RNN-140402 25159870 PMC4524647

[17] Lidzba K de Haan B Wilke M Krageloh-Mann I Staudt M. Lesion characteristics driving right-hemispheric language reorganization in congenital left-hemispheric brain damage. Brain Lang. (2017a) 173:1–9. 10.1016/j.bandl.2017.04.006 28549234

[18] deVeber G Roach ES Riela AR Wiznitzer M. Stroke in children: recognition, treatment, and future directions. Semin Pediatr Neurol. (2000) 7(4):309–17. 10.1053/spen.2000.20074 11205720

[19] Gao L Lim M Nguyen D Bowe S MacKay MT Stojanovski B The incidence of pediatric ischemic stroke: a systematic review and meta-analysis. Int J Stroke. (2023) 18(7):765–72. 10.1177/17474930231155336 36691675

[20] Westmacott R Askalan R Macgregor D Anderson P Deveber G. Cognitive outcome following unilateral arterial ischaemic stroke in childhood: effects of age at stroke and lesion location. Dev Med Child Neurol. (2010) 52(4):386–93. 10.1111/j.1469-8749.2009.03403.x 19694778

[21] Studer M Boltshauser E Capone Mori A Datta A Fluss J Mercati D Factors affecting cognitive outcome in early pediatric stroke. Neurology. (2014) 82(9):784–92. 10.1212/WNL.0000000000000162 24489131

[22] Fuentes A Deotto A Desrocher M deVeber G Westmacott R. Determinants of cognitive outcomes of perinatal and childhood stroke: a review. Child Neuropsychol. (2016) 22(1):1–38. 10.1080/09297049.2014.969694 25355013

[23] Gilardone G Viganò M Cassinelli D Fumagalli FM Calvo I Gilardone M Post-stroke acquired childhood aphasia. A scoping review. Child Neuropsychol. (2023) 29(8):1268–93. 10.1080/09297049.2022.2156992 36548197

[24] Lidzba K Küpper H Kluger G Staudt M. The time window for successful right-hemispheric language reorganization in children. Eur J Paediatr Neurol. (2017b) 21(5):715–21. 10.1016/j.ejpn.2017.06.001 28648758

[25] Goeggel Simonetti B Cavelti A Arnold M Bigi S Regényi M Mattle HP Long-term outcome after arterial ischemic stroke in children and young adults. Neurology. (2015) 84(19):1941–7. 10.1212/WNL.0000000000001555 25862797

[26] Lidzba K Bürki SE Staudt M. Predicting language outcome after left hemispherotomy: a systematic literature review. Neurol Clin Pract. (2021) 11(2):158–66. 10.1212/CPJ.0000000000000852 33842069 PMC8032409

[27] Rorden C Karnath HO Bonilha L. Improving lesion-symptom mapping. J Cogn Neurosci. (2007) 19(7):1081–8. 10.1162/jocn.2007.19.7.1081 17583985

[28] Wilke M Lidzba K Staudt M Buchenau K Grodd W Krageloh-Mann I. An fMRI task battery for assessing hemispheric language dominance in children. Neuroimage. (2006) 32(1):400–10. 10.1016/j.neuroimage.2006.03.012 16651012

[29] Meinhold T Hofer W Pieper T Kudernatsch M Staudt M. Presurgical language fMRI in children, adolescents and young adults. Clin Neuroradiol. (2020) 30(4):691–704. 10.1007/s00062-019-00852-7 31960077

[30] Schnaufer L Gschaidmeier A Heimgärtner M Driever PH Hauser T-K Wilke M Atypical language organization following perinatal infarctions of the left hemisphere is associated with structural changes in right-hemispheric grey matter. Dev Med Child Neurol. (2024a) 66(3):353–61. 10.1111/dmcn.15751 37691416

[31] Dunn LM Dunn DM. PPVT-4: Peabody picture vocabulary test, Pearson Assessments (2007).

[32] Esser Gn Wyschkon A. (2010). P-IPTA deutsche Fassung des Illinois test of psycholinguistic abilities, 3rd ed (ITPA-3) von D.D. Hammill, N. Mather & R. Roberts: Manual.

[33] Brown L Sherbenou RJ Johnsen SK. Test of nonverbal intelligence: TONI-4, Pro-ed Austin. TX. (2010).

[34] Staudt M Lidzba K Grodd W Wildgruber D Erb M Krageloh-Mann I. Right-hemispheric organization of language following early left-sided brain lesions: functional MRI topography. Neuroimage. (2002) 16(4):954–67. 10.1006/nimg.2002.1108 12202083

[35] Staudt M. Reorganization after pre- and perinatal brain lesions*. J Anat. (2010) 217(4):469–74. 10.1111/j.1469-7580.2010.01262.x 20649910 PMC2992421

[36] Tsouli S Kyritsis AP Tsagalis G Virvidaki E Vemmos KN. Significance of aphasia after first-ever acute stroke: impact on early and late outcomes. Neuroepidemiology. (2009) 33(2):96–102. 10.1159/000222091 19494550

[37] Greenham M Anderson V Mackay MT. Improving cognitive outcomes for pediatric stroke. Curr Opin Neurol. (2017) 30(2):127–32. 10.1097/WCO.0000000000000422 28141739

[38] Lõo S Ilves P Männamaa M Laugesaar R Loorits D Tomberg T Long-term neurodevelopmental outcome after perinatal arterial ischemic stroke and periventricular venous infarction. Eur J Paediatr Neurol. (2018) 22(6):1006–15. 10.1016/j.ejpn.2018.07.005 30249407

[39] Dunbar M Kirton A. Perinatal stroke. Semin Pediatr Neurol. (2019) 32:100767. 10.1016/j.spen.2019.08.003 31813521

[40] O'Hare A. Management of developmental speech and language disorders. Part 2: acquired conditions. Arch Dis Child. (2016) 101(3):278–83. 10.1136/archdischild-2014-306153 25990500

[41] Westmacott R MacGregor D Askalan R deVeber G. Late emergence of cognitive deficits after unilateral neonatal stroke. Stroke. (2009) 40(6):2012–9. 10.1161/STROKEAHA.108.533976 19423855

[42] Guglielmi RS. Native language proficiency, English literacy, academic achievement, and occupational attainment in limited-English-proficient students: a latent growth modeling perspective. J Educ Psychol. (2008) 100(2):322–42. 10.1037/0022-0663.100.2.322

[43] Sylvestre A Brisson J Lepage C Nadeau L Deaudelin I. Social participation of children age 8–12 with SLI. Disabil Rehabil. (2016) 38(12):1146–56. 10.3109/09638288.2015.1074730 26287388

[44] van Buuren LM van der Aa NE Dekker HC Vermeulen RJ van Nieuwenhuizen O van Schooneveld MM Cognitive outcome in childhood after unilateral perinatal brain injury. Dev Med Child Neurol. (2013) 55(10):934–40. 10.1111/dmcn.12187 23758403

