# Supplementary material for: The long-term negative impact of childhood stroke on language
Source: Front Pediatr. 2024 May 7;12:1338855. doi: 10.3389/fped.2024.1338855 (PMC11106365; doi:10.3389/fped.2024.1338855)
Supplement: Supplementary file 1 [file Datasheet1.pdf]

Supplementary Material

Longterm Negative Impact of Childhood Stroke on Language

Magdalena Heimgärtner, Alisa Gschaidmeier, Lukas Schnaufer, Martin Staudt, Marko Wilke, Karen Lidzba

Post-hoc Analyses and Results

To better understand the divergent results between this study and a previous publication on a partly identical patient group (Gschaidmeier, Heimgärtner et al. 2022), we conducted the following additional post-hoc analyses separately for patients younger (CHILD: n = 4; PERI: n = 11; CONTROL: n = 16) and older than 12 years (CHILD: n = 5; PERI: n = 12; CONTROL: n = 17):

1) Effect on "language score". We conducted two univariate analyses of covariance (ANCOVA), with the factor group (CHILD vs. PERI vs. CONTROL), the independent variable "language score", and the covariate IQ (TONI-4 z-score).

1a) Subgroup of patients < 12 years. "Language Score" equals the P-ITPA sample-standardized z-score. After correcting for the significant effect of IQ, there is no significant group effect ($F_{2,31} = 0.742$, $p = .485$).

1b) Subgroup of patients > 12 years. "Language Score" equals the PPVT sample-standardized z-score. After correcting for the significant effect of IQ, there is a significant group effect ($F_{2,34} = 10.676$, $p < .001$). Post-hoc analyses reveal that both patient groups perform significant below the control groups.

2) Effect on P-ITPA / PPVT raw scores.

2a) Subgroup of patients < 12 years. We conducted a univariate analysis of covariance (ANCOVA), with the factor group (CHILD vs. PERI vs. CONTROL), the independent variable PPVT raw score, and the covariate IQ (TONI-4 z-score). After correcting for the significant effect of IQ, there is no significant group effect ($F_{2,31} = 0.462$, $p = .635$).

2b) Subgroup of patients > 12 years. We conducted a univariate analysis of covariance (ANCOVA), with the factor group (CHILD vs. PERI vs. CONTROL), the independent variable P-ITPA raw score, and the covariate IQ (TONI-4 z-score). After correcting for the significant

effect of IQ, there is a significant group effect ($F_{2,34} = 20.055$, $p < .001$). Post-hoc analyses reveal that both patient groups perform significant below the control group, and that the CHILD group performed significantly better than the PERI group.

Discussion

Both analyses for the younger vs. older age groups reveal significant group effects for the older, but not the younger participants, independently of the tests used to operationalize language function. Both with the age-appropriate standard scores of the PPVT, and with the raw scores of the P-ITPA, the older groups of patients with both perinatal and childhood strokes perform significantly below the control groups. In the younger sample, using the standard scores of the P-ITPA and the raw scores of the PPVT, this effect is not visible at all. Although the small sample size (especially of group POST) limits the generalizability of this result, it is apt to explain the diverging results between our two studies.
